# A novel strategy for efficient production of anti-V3 human scFvs against HIV-1 clade C

**DOI:** 10.1186/1472-6750-12-87

**Published:** 2012-11-15

**Authors:** Rajesh Kumar, Raiees Andrabi, Ashutosh Tiwari, Somi Sankaran Prakash, Naveet Wig, Durgashree Dutta, Anurag Sankhyan, Lubina Khan, Subrata Sinha, Kalpana Luthra

**Affiliations:** 1 Department of Biochemistry, All India Institute of Medical Sciences, New Delhi, India; 2 Department of Medicine, All India Institute of Medical Sciences, New Delhi, India; 3 National Brain Research Centre, Manesar, India; 4 Present address: Centre for Biodesign, Translational Health Science and Technology Institute, Gurgaon, India

**Keywords:** HIV-1, Clade C, V3, scFv

## Abstract

**Background:**

Production of human monoclonal antibodies that exhibit broadly neutralizing activity is needed for preventing HIV-1 infection, however only a few such antibodies have been generated till date. Isolation of antibodies by the hybridoma technology is a cumbersome process with fewer yields. Further, the loss of unstable or slowly growing clones which may have unique binding specificities often occurs during cloning and propagation and the strongly positive clones are often lost. This has been avoided by the process described in this paper, wherein, by combining the strategy of EBV transformation and recombinant DNA technology, we constructed human single chain variable fragments (scFvs) against the third variable region (V3) of the clade C HIV-1 envelope.

**Results:**

An antigen specific phage library of 7000 clones was constructed from the enriched V3- positive antibody secreting EBV transformed cells. By ligation of the digested scFv DNA into phagemid vector and bio panning against the HIV-1 consensus C and B V3 peptides followed by random selection of 40 clones, we identified 15 clones that showed V3 reactivity in phage ELISA. DNA fingerprinting analysis and sequencing showed that 13 out of the 15 clones were distinct. Expression of the positive clones was tested by SDS-PAGE and Western blot. All the 13 anti-V3 scFvs showed cross-reactivity against both the clade C and B V3 peptides and did not show any reactivity against other unrelated peptides in ELISA. Preliminary neutralization assays indicated varying degrees of neutralization of clade C and B viruses. EBV transformation, followed by antigen selection of lines to identify specific binders, enabled the selection of phage from un-cloned lines for scFv generation, thus avoiding the problems of hybridoma technology. Moreover, as the clones were pretested for antigen binding, a comparatively small library sufficed for the selection of a considerable number of unique antigen binding phage. After selection, the phage clones were propagated in a clonal manner.

**Conclusions:**

This strategy can be efficiently used and is cost effective for the generation of diverse recombinant antibodies. This is the first study to generate anti-V3 scFvs against HIV-1 Clade C.

## Background

There is a rapid increase in the number of human immunodeficiency virus (HIV-1) infected individuals worldwide and so far we have met with little success in slowing down or preventing the progression of this pandemic disease. In order to use broadly neutralizing antibodies as effective reagents for passive immunotherapy to slow or to halt the disease progression in HIV-1 infected individuals and for immunogen design for vaccination to prevent the infection, the generation of large numbers of human HIV-1 specific monoclonal antibodies is desirable. Although a few human broadly neutralizing antibodies (bNAbs) to HIV-1 exist [1-10], these antibodies have limited reactivity against non-clade B viruses, which are responsible for more than 85% of the infections worldwide [4]. Few bNAbs exist so far, that are effective against the clade C viruses, which include the 4E10, antibodies from the CAPRISA cohort and the recently isolated monoclonal antibodies PG9, PG16 and VRC01 [9-12] . In order to evaluate their utility in combating HIV-1 infection, and to tackle the problems posed by the extensive diversity of HIV-1, it is essential to generate a large panel of human anti-HIV-1 antibodies of different specificities. Further, it may be necessary to evaluate several antibodies to find rare but highly effective molecules.

The methods used for the generation of human monoclonal antibodies include the hybridoma technology, recombinant technology by phage display and the recently employed techniques such as single B cell sorting followed by amplification of heavy and light chain genes [8,13,14]. Generation of antibodies by the conventional hybridoma technology is not adequate enough to meet the challenge of assessing large numbers of human monoclonal antibodies from HIV-1 infected individuals at various stages of their clinical course. Our approach to the problem has been to combine the antigen specific pre-selection of EBV transformed B cells with the construction of a phage library.

Phage display is a scalable method for antibody production against a wide variety of antigens [15-17]. Investigators are using this technology for the production of antibodies with the desired isotype specificities and affinity for research, clinical and industrial applications. Antibody gene variable regions are amplified and expressed on the surface of filamentous bacteriophage as a fusion protein [14] and a number of antibodies can be produced from a single library and can be expressed and produced in a prokaryotic system. The major drawback in this technology is the requirement for the construction of large sized phage libraries (10^9^-10^10^) with an optimum diversity because the diversity of these libraries primarily determines the successful isolation of the desired antibodies. Construction of such large libraries requires a number of ligation and electroporation reactions. Screening of positive clones from these libraries generally require at least three to seven rounds of biopanning to select specifically binding clones [18,19]. It is also difficult to maintain such large sized libraries. Keeping all these factors in mind we have described here a simple and efficient method for the production of human monoclonal antibodies using a combinational approach of EBV transformation, antigen preselection and phage display technology. A similar approach was previously used by Kempf et al. to isolate Fabs to the gp120 of HIV-1 [20]. Here, we adopted a modified strategy and isolated for the first time, human anti-V3 scFvs from the EBV transformed lymphocytes of a clade C HIV-1 infected patient. This method can be used to produce large panels of viral specific phage libraries in less time and in a cost effective manner.

A limited number of monoclonal antibodies are generated against the HIV-1 Clade C which is the most predominant subtype worldwide and in India [21]. The V3 region of HIV-1 gp120 is an important target to induce neutralizing antibodies against different strains of HIV-1. It exhibits a high degree of sequence variability in the most structurally conserved region of the HIV-1 envelope [22,23] and it interacts with the co-receptors CCR5 and CXCR4 [7,24,25]. In this study, we constructed a scFv library from the EBV transformed B cells of an HIV-1 infected patient #254, reactive to the third variable (V3) region of gp120 of HIV-1 clade C. The plasma sample of this patient was previously tested and found to exhibit cross neutralizing activity against a standard panel of pseudoviruses of different clades (Table 1) and also had cross reactive binding antibodies to clade C and B V3 peptides [26,27]. The modified strategy used here employing EBV transformation; antigen preselection followed by phage library construction enabled the successful selection of human monoclonal recombinant scFvs specific to the V3 antigen of HIV-1. Construction of human scFv phage library from these antigen preselected B cells led to an efficient and cost effective approach for the production of anti-V3 scFvs.

**Table 1:** Neutralization (ID50) of subtype-A, B and C viruses by #254 plasma sample

**Sr. No.**	**Virus**	**Tier**	**Subtype**	**ID50**
**1**	92RW009	1	A	*74*
**2**	Q461	1	A	*98*
**3**	SF162	1	B	**1296**
**4**	JRCSF	1	B	***103***
**5**	Du156.12	2	C	***206***
**6**	ZM53M.PB12	2	C	<60
**7**	ZM109F.PB4	2	C	***104***
**8**	JRFL	2	B	<60
**9**	RHPA4259.7	2	B	***446***
**10**	TRO.11	2	B	***162***

The cross neutralizing activities of plasma antibodies from HIV-1 infected drug naïve sample #254 against a panel of subtype-A, B, C tier 1 and 2 viruses indicated on left. The numerical values on the extreme right of table represent the mean 50% neutralization titers (ID50) defined as the reciprocal dilution of plasma which neutralized 50% of viral infection in the assay. For clarity, this information is coded; ID50> 1000 (Bold), ID50= 100-l000 (Bold Italic), ID50= 61–100 (Italic) and ID50<60, where 50% neutralization was not reached. Each experiment was performed at least two independent times and in duplicates.

## Results

### Viral neutralization and cross reactive binding activity of the patient plasma #254

**Figure 1:**
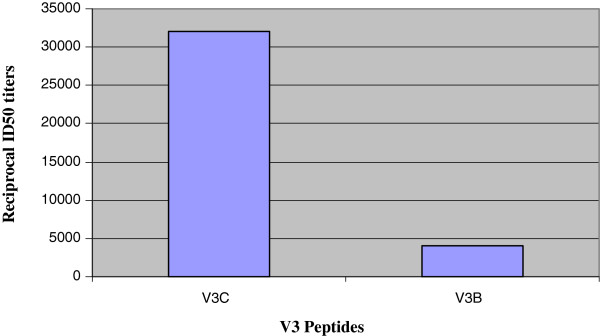
**Level of Anti V3 antibodies in patient plasma (#254).** 50% reciprocal binding titer of the plasma of the patient (#254) against consensus clade C and B V3 peptides. The results depict the average OD values of three independent experiments.

The plasma antibodies of the HIV-1 infected patient #254 displayed neutralizing activity against 8 out of 10 HIV-1 viruses from subtype-A, B and C (Table 1). The plasma sample also showed a cross subtype-B and C binding reactivity in context of the anti-V3 loop directed antibodies (Figure 1).

### EBV transformation

**Figure 2:**
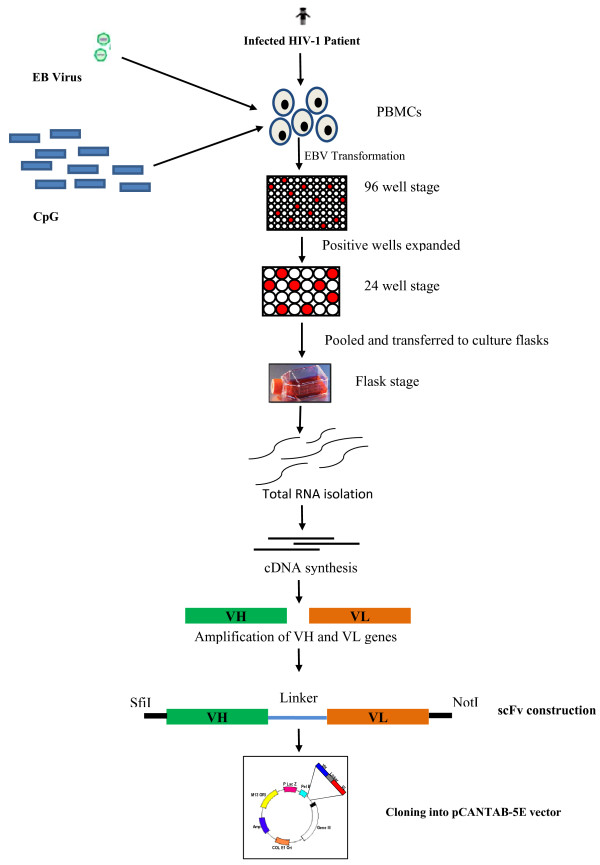
**Schematic overview of the strategy used for construction of antigen specific phage library.** The peripheral blood mononuclear cells (PBMCs) were isolated from an antiretroviral drug naïve patient (#254) whose plasma exhibited neutralizing activity against a panel of viruses and also displayed cross clade reactive anti-V3 antibody binding potential. PBMCs were subjected to EBV transformation in 96 well culture plate, wells showing high level of anti-V3 antibodies were selected and expanded to 24 well stage and then to six well stage . The wells showing high titre of anti-V3 Abs were pooled together and cultured in T25 flask. Total RNA was isolated from these antigen specific enriched B cells and cDNA was synthesised. Heavy and light chains were amplified and scFvs were constructed and cloned into a pCANTAB-5E phagemid vector. scFv phage library of 7000 clones was constructed.

A total of 4.8 million PBMCs were isolated from the HIV-infected patient (# 254) and EBV transformed in 48 wells of a 96 well culture plate (Figure 2). After 2 weeks, the cultures were screened (first screening) for the presence of anti-V3 antibodies and 7/48 wells showed high V3 binding reactivity with OD> 2. Cells from these 7 V3 positive wells were expanded to a 24 well plate and after a second screening, cells from 5 out of 7 wells showing V3 binding with OD > 0.6 were expanded in a six-well plate, pooled and finally transferred to a T- 25 flask. In the 3rd (final) screening of the cells in the flask, the cells showed high reactivity to V3 (OD > 1) (Table 2). These V3 positive (enriched) cells were processed for recombinant anti-V3 scFv generation.

**Table 2:** Screening of EBV transformed PBMCs isolated from a drug naïve HIV-1 infected patient (# 254) for V3 reactivity

^1^Total number of PBMCs isolated (millions) from HIV-1 infected patient # 254	4.8
^2^Number of cells plated per well	100000
^3^Total number of wells plated with the cells and EBV transformed in 96 well culture plate	48
^4^Number of wells successfully EBV transformed	48
^5^Number of wells secreting anti-V3 Abs (OD>2) in 96 well stage(first screening)	7
^6^Number of wells secreting anti-V3 Abs (OD>0.6) in 24 well stage(second screening)	5
^7^OD at flask stage(Final screening) (OD>1.0)	

^1^ PBMCs were isolated from a HIV positive drug naïve patient sample **(# 254)**. A total of 4.8 million PBMCs were isolated.

^2^ Number of cells plated per well was 100000.

^3^ Forty eight wells were subjected to EBV transformation.

^4^ All the 48 wells were successfully transformed (transformation efficiency 100%).

^5^ 7/48(approx 14.6%) wells showed positive binding (OD>2) with V3 peptide and expanded to 24 well stage.

^6^ 5/7 (approx 71%) wells showed positive binding (OD>0.6) with V3 peptide and pooled down and grown in T-25 flask.

^7^ Final screening for anti-V3 antibody binding reactivity was done at flask stage (OD>1.0).

### Phage library construction

**Figure 3:**
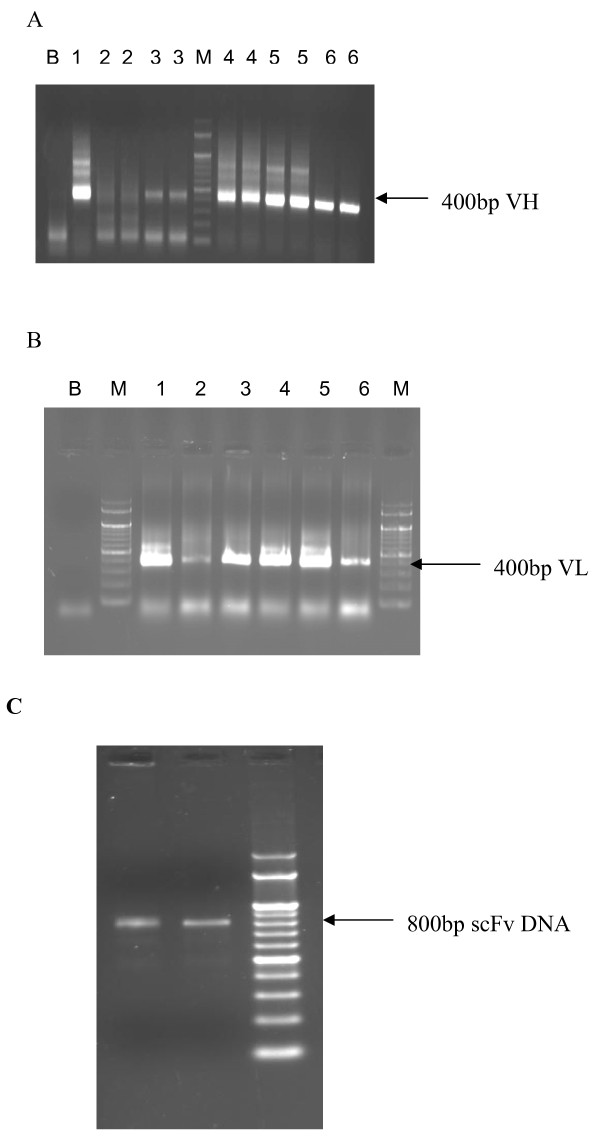
**Amplification of VH and VL genes. A**. Agarose gel electrophoresis (1%) of PCR amplified products of heavy chain (VH) genes using all combinations of the 24 primers. Lane B, PCR blank (negative control); Lane M, DNA marker (100 bp ladder); Lanes 1–6 is with VH1-6 with reverse primers (R1, R2, R3 and R4). Samples that were loaded in duplicates are indicated as numbers 2, 2 and 3, 3 so on. **B**. Agarose gel electrophoresis (1%) of PCR amplified products of light chain (VL) genes. Lane B, PCR blank negative control; Lane M, DNA marker (100 bp ladder); Lane 1–6 is with VKL1-6 with reverse primers. **C**. Pull through PCR for scFv construction**,** Agarose gel electrophoresis (1%) of PCR amplified products of scFvs constructed by pull through PCR. Lane M, DNA ladder (100 bp); Lanes 1 & 2 are amplified scFv DNA products.

Total RNA from the V3 enriched antibody producing B cells of the patient #254 was isolated. A total of 200ng of RNA was reverse transcribed to cDNA. Use of random hexamer and oligo dT in the cDNA synthesis process was shown to be more effective than either of them alone or the 3^′^ primers [15,20]. It also enhanced the chances of amplification of the antibody genes even when the amount of RNA is low [15]. A total of 54 combinations were used to amplify the heavy and light chains (24 for VH and 30 for VL). Among the 24 combinations of heavy chains, 18 were successfully amplified. The heavy chains VH1, VH4, VH5 and VH6 were preferentially more expressed while VH2 and VH3 were very less expressed (Figure 3A). All the 30 kappa light chain combinations were successfully amplified and expressed almost at the same level, except for VLK2, which were relatively less expressed (Figure 3B). A heavy chain and light chain pool was made by mixing equal concentration of each combination (50ng) irrespective of their expression level, so that each combination of the heavy chain has an equal probability to combine with the light chain and vice versa and to avoid the predominant expression of one antibody subclass and finally to have new combinations of antibodies for a particular antigen that are naturally not elicited during the natural course of infection. A final pull through PCR was performed using the forward and reverse primers. These scFvs were then ligated into the pCANTAB-5E vector that contains the *Sfi*1 and *Not*I sites (Figure 3). The ligated product was transformed into TG1 using calcium chloride mediated transformation. The incubation time after the heat shock treatment during transformation was maintained for a maximum of 40 min to avoid repetition of similar types of clones in the library. We obtained 200–300 transformed colonies per plate. A total of 7000 colonies were obtained with 400ng of digested scFv DNA that were ligated into 1 μg of phagemid vector. The ligated DNA was transformed into *E.coli* TG1.

### Diversity of the phage antibody library

**Figure 4:**
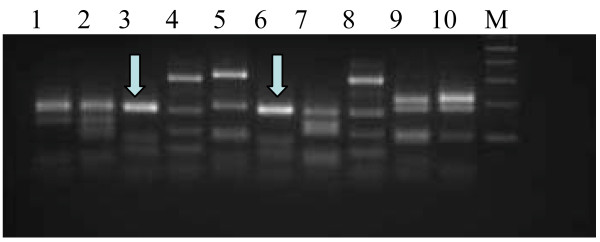
**DNA fingerprinting analysis of scFv clones.** Ten scFv gene fragments were amplified by PCR and digested with B*stN*1 at 60°C for 3 hours. Restriction pattern was analysed on 2% agarose gel. Arrows indicate that clones 3 and 6 exhibit identical DNA fingerprinting pattern. Lane M, DNA ladder (100 bp).

To check the diversity of antibodies in the library, we randomly selected 10 clones from the unselected library. Amplification of these 10 scFvs by PCR, followed by digestion with *BstN1* and comparing their DNA fingerprint patterns showed that 9/10 clones were distinct from each other. Clone 3 and clone 6 showed identical DNA fingerprinting patterns (Figure 4).

We further confirmed the diversity of the antibody fragments by DNA sequence analysis of 29 randomly selected scFv clones from the unselected library which showed high diversity of clones i.e. 73% (21/29) in the library. The heavy chain (VH) sequences showed a limited diversity, IGVH4 (13/29) and IGVH5 (15/29) were the most preferentially represented VH sequences that combined with all the representatives of IGHD genes except for IGHD1. Diversity of the VL paratope was also limited with IGKV1, IGKV2 and IGKV3 being most represented, except for one occurrence of IGKV7 (Table 3).

**Table 3:** Diversity of scFvs in unselected phage library

	**VH**			**VL**	
**scFv**	**V**	**D**	**J**	**V**	**J**
**1**	IGHV1-18*01	IGHD6-19*01	IGHJ3*02	IGKV3-NL5*01	IGKJ2*02
**2**	IGHV4-b*02	IGHD3-10*02	IGHJ4*02	IGKV3-20*01	IGKJ2*01
**3**	IGHV4-b*02	IGHD3-3*01	IGHJ6*02	IGKV1D-12*02	IGKJ3*01
**4**	IGHV4-b*02	IGHD3-10*02	IGHJ4*02	IGKV2D-28*01	IGKJ2*01
**5**	IGHV4-b*02	IGHD3-10*02	IGHJ4*02	IGKV3-11*01	IGKJ1*01
**6**	IGHV4-b*02	IGHD3-10*02	IGHJ4*02	IGKV3-20*01	IGKJ4*01
**7**	IGHV4-b*02	IGHD3-10*02	IGHJ4*02	IGKV2-30*01	IGKJ4*01
**8**	IGHV4-b*02	IGHD4-4*01	IGHJ5*02	IGKV2D-28*01	IGKJ2*01
**9**	IGHV4-b*02	IGHD6-6*01	IGHJ3P*01	IGHV1/OR15-2*02	IGHD5-5*01
**10**	IGHV4-b*02	IGHD5-24*01	IGHJ4*02	IGKV3-20*01	IGKJ2*01
**11**	IGHV4-31*03	IGHD3-10*02	IGHJ4*02	IGKV3-20*01	IGKJ2*01
**12**	IGHV4-31*03	IGHD5-5*01	IGHJ6*03	IGKV1D-39*01	IGKJ1*01
**13**	IGHV4-61*02	IGHD2-8*02	IGHJ3*02	IGKV1D-12*02	IGKJ2*01
**14**	IGHV4-59*08	IGHD5-24*01	IGHJ3*02	IGKV3D-20*01	IGKJ1*01
15	IGHV5-51*01	IGHD4-4*01	IGHJ6*01	IGKV2D-28*01	IGKJ2*01
16	IGHV5-51*01	IGHD4-4*01	IGHJ6*01	IGKV2D-28*01	IGKJ2*01
**17**	IGHV5-51*01	IGHD4-4*01	IGHJ5*02	IGKV2D-28*01	IGKJ2*01
**18**	IGHV5-51*01	IGHD4-4*01	IGHJ5*02	IGKV2D-28*01	IGKJ2*01
**19**	IGHV5-51*01	IGHD4-4*01	IGHJ5*02	IGKV2D-28*01	IGKJ2*01
**20**	IGHV5-51*01	IGHD4-4*01	IGHJ5*02	IGKV2-30*02	IGKJ1*01
**21**	IGHV5-51*01	IGHD4-4*01	IGHJ5*02	IGKV1D-12*02	IGKJ3*01
**22**	IGHV5-51*01	IGHD4-4*01	IGHJ5*02	IGKV1D-12*02	IGKJ3*01
**23**	IGHV5-51*01	IGHD4-4*01	IGHJ5*02	IGKV1D-12*02	IGKJ3*01
**24**	IGHV5-51*01	IGHD4-4*01	IGHJ5*02	IGKV1D-12*02	IGKJ3*01
**25**	IGHV5-51*01	IGHD4-4*01	IGHJ5*02	IGKV3-20*01	IGKJ3*01
**26**	IGHV5-51*01	IGHD4-4*01	IGHJ5*02	IGKV3-20*01	IGKJ1*01
**27**	IGHV5-51*01	IGHD4-4*01	IGHJ5*02	IGKV7-3*01	IGKJ2*04
**28**	IGHV5-51*01	IGHD4-4*01	IGHJ5*02	IGKV3-11*02	IGKJ1*01
**29**	IGHV5-51*01	IGHD4-4*01	IGHJ5*02	IGKV3-11*01	IGKJ1*01

Identical sequences of clones 15 and 16, 18 and 19, 22, 23 and 24 are shown in italic.

### Selection of scFvs against the V3 region of HIV-1

**Figure 5:**
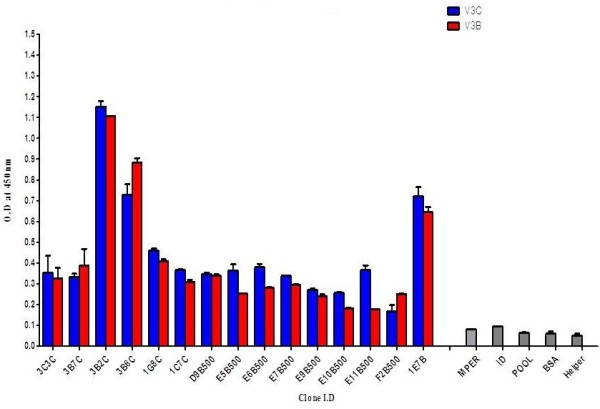
**Phage ELISA binding specificity.** Selection of clones exhibiting binding to the V3 peptides of clade C and clade B HIV-1. ELISA wells were coated with the V3C and V3B peptide. MPER peptide, ID loop peptide, BSA and a peptide pool of unrelated viruses were used as negative controls. The experiment was done in duplicates and repeated at least twice and the mean OD values are shown. Clones showing O.D three times the negative control was considered as positive.

As the size of the library was very small, one round of biopanning was done against the V3 C and V3 B peptides. Forty clones were randomly selected and checked for their binding, of which 15 showed positivity in phage ELISA with both the peptides (Figure 5). Four clones were randomly selected for ELISA with purified phage. The binding specificity of these anti-V3 scFvs was checked with phage from the 24 well plate and PEG precipitated purified phage. The 24 well ELISA and pure phage ELISA binding assays showed similar results. Three clones exhibited almost similar binding in both the formats of ELISA while one clone had lower binding reactivity in pure phage ELISA although it was not completely lost (data not shown). DNA fingerprinting analysis using *BstN1* followed by sequencing revealed that 13/15 clones were distinct (Table 4). All the distinct 13 anti -V3 scFvs that were finally selected, showed cross-reactivity against both the V3 peptides and did not show any reactivity against other unrelated peptides. One round of biopanning was found to be sufficient to get V3 positive scFv clones with diversity.

**Table 4:** Gene usage of anti-V3 scFvs

**scFv**	**VH**			**VL**	
	**V**	**D**	**J**	**V**	**J**
**1D5C**	IGHV3-23*04	IGHD3-10*01	IGHJ3*02	IGKV3-20*01	IGKJ1*01
**1E7B**	IGHV4-31*03	IGHD5-24*01	IGHJ6*03	IGKV3-20*01	IGKJ2*01
**1G8C**	IGHV5-51*01	IGHD4-11*01	IGHJ5*02	IGKV2-28*01	IGKJ2*01
**3B2C**	IGHV5-51*01	IGHD4-11*01	IGHJ5*02	IGKV1-5*03	IGKJ4*01
**3B6C**	IGHV4-b*02	IGHD3-10*02	IGHJ4*02	IGKV1-39*01	IGKJ2*02
**3B7C**	IGHV4-b*02	IGHD3-10*02	IGHJ1*01	IGKV3-20*01	IGKJ4*01
***3C3C***	IGHV5-51*01	IGHD4-11*01	IGHJ5*02	IGKV3-20*01	IGKJ1*01
**3E6C**	IGHV4-59*08	IGHD3-10*02	IGHJ1*01	IGKV1-12*02	IGKJ3*01
**3E6B**	IGHV4-b*02	IGHD3-10*02	IGHJ4*02	IGKV1-39*01	IGKJ4*01
***3E7B***	IGHV5-51*01	IGHD4-11*01	IGHJ5*02	IGKV3-20*01	IGKJ1*01

This table shows the gene usage of the ten anti-V3 scFvs. Nine out of the ten sequences are distinct. Clones 3C3C and 3E7B show similar gene usage which are shown in italic.

### Soluble scFv production

**Figure 6:**
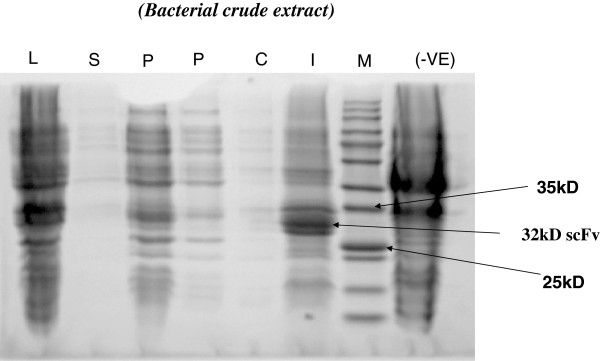
**Localization of scFv antibody in different fractions.** Lane M, protein molecular weight marker ; lane L, cell lysate fraction; lane S, culture supernatant; lane P1 and P2 are, periplasmic fraction (20 μl and 10 μl respectively loaded); lane I, inclusion body (insoluble) fraction. Lane –Ve control, cell lysate of phagemid vector without scFv was used.

The antigen binding clones showing positivity/binding in the phage ELISA were further processed for soluble scFv expression by induction with 1mM IPTG [28]. After induction, the periplasmic lysate, inclusion bodies, culture supernatant and whole cell extract were prepared and analysed for scFv expression on a 12% reducing SDS-PAGE. The scFv fusion protein was found to be expressed in the cell lysate, culture supernatant, periplasmic extract, with highest expression in the inclusion bodies. scFv expression was also observed in the uninduced culture (Figure 6).

### Purification of E-tagged scFv and Western blot analysis

**Figure 7:**
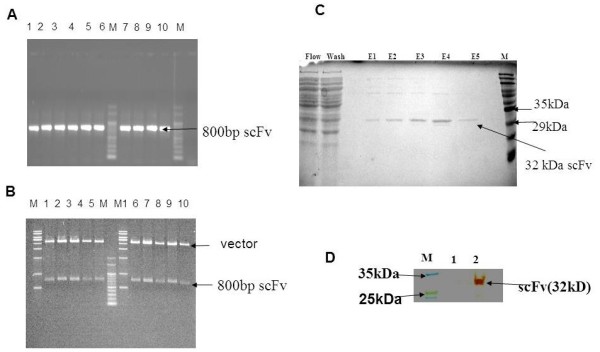
**Analysis of scFv clones by agarose gel electrophoresis (1%) and purification of scFvs. A**. Agarose gel analysis of colony PCR of 10 randomly selected clones from library before panning. **B**. Agarose gel analysis of *Sfi*I and *Not*I digested plasmid of 10 randomly selected clones from library before panning. Lane M, 100 bp marker; Lane M1 1kb ladder; Lane 1–10 are plasmid DNA from ten different clones. **C**. SDS- PAGE (12 %) of purified scFv Lane M, protein marker; Lane E1 to E5, different eluted fractions of purified scFv. Arrow indicates the 32kDa band of scFv in E1 to E5 lanes. **D**. Western blot analysis of purified scFv, pCANTAB-5E vector periplasmic extract was used as a negative control. Lane M, prestained molecular weight marker.

The scFvs were expressed in *E. coli* HB2151 cells by inducing for 6 to 8 h with 1 mM IPTG at 24°C. The antibody fragments were purified from the periplasmic extract and the purified product was analysed by SDS-PAGE. The scFv protein of 32kDa was expressed in different elute fractions E1 to E5 (Figure 7C) and protein samples were concentrated using ultrafiltration columns (Ambion) over a 10kDa cut off.

Expression of the scFv fusion protein (32 kD) in the periplasmic extract was confirmed by Western blotting. The cell lysate of HB2151 was used as a negative control (Figure 7D).

### Validation of antigen binding of the scFv clones

**Figure 8:**
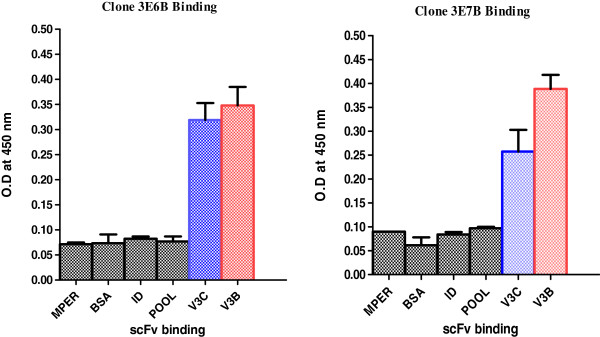
**Binding specificity of selected anti-V3 scFv clones.** The binding of scFv antibodies was determined by ELISA against V3 peptides of clade C and B, MPER and ID peptides of HIV, peptide pool (unrelated viruses) and BSA. The clones 3E6B and 3E7B bound specifically to V3 peptides. The experiment was performed in triplicates.

The functional activity of the newly generated clones 3E6B and 3E7B was assessed by their binding to the V3 peptides of Clade C and B in ELISA. Both the scFvs showed specific binding to the V3 peptides (Figure 8). In addition, these scFvs did not bind to other unrelated peptides like MPER and ID loop of HIV, peptide pool (unrelated viruses) and BSA.

### Neutralization potential of anti-V3 scFvs

The purified scFvs were tested for the viral neutralization potential against a panel of pseudoviruses from clades A, B and C HIV-1 viruses (Table 5). Clone 3E6B was able to neutralize 1/1 clade A, 2/2 clade B and 1/3 clade C viruses. The other clone 3E7B was only able to neutralize 1 clade C virus.

**Table 5:** Neutralization potential of anti-V3 scFvs against a panel of pseudoviruses and primary isolates

**Virus**	**Clade**	**3E6B**	**3E7B**	**scFv (HepB)**	**1418**
**92RW**	**A**	**45**	**>100**	**>150**	**>150**
**SF162**	**B**	**115**	**>100**	**>150**	**>150**
**QZ4589**	**B**	**50**	**>100**	**>150**	**>150**
**AIIMS 261**	**C**	**110**	**45**	**>150**	**>150**
**Du 422**	**C**	**>150**	**>100**	**>150**	**>150**
**ZM 53**	**C**	**>150**	**>100**	**>150**	**>150**

The cross neutralizing activities of two anti-V3 scFv clones against a panel of subtype-A, B, C viruses that are listed on the left. The numerical values in the table represent the concentration (μg/ml) of scFvs at which 50% neutralization titers (IC50) was reached. AIIMS 261 is the primary isolate generated in our lab from a HIV-1 clade C infected patient. scFv (HepB) is an scFv against the Hepatitis B surface antigen (negative control) and 1418 is human antibody to parvovirus (negative control). Each experiment was performed at least two independent times.

## Discussion

We have demonstrated in this study a strategy that successfully yielded recombinant human scFvs against the V3 region of HIV-1 using a pool of antigen selected EBV transformed B lymphocytes and phage display technology. The use of preselected B cells producing anti- V3 antibodies for phage scFv library construction proved to be an efficient strategy for the isolation of V3 specific clones. Moreover, this approach has prevented any loss of unstable or slowly growing clones which may have useful and unique binding specificities (that often occurs during cloning and propagation).

Stringency during the initial stages helps in elimination of most of the false positive clones. Beginning with the 96 well stage and up to the final screening of the cells in the T25 flask, we selected only those wells with high V3 binding reactivity for further expansion and finally for the phage library construction. By this approach, we obtained a phage library with an adequate number of V3 binding clones. Amplification of the heavy chain gene showed that the VH1, VH4, VH5 and VH6 were preferentially more expressed than the VH2 and VH3 and corroborated with the previous reports [29] that anti-V3 antibodies more preferentially exhibit the above gene usages. It is well established that in HIV-1 infection, VH3 genes are less preferentially used [30,31]. From the sequencing data, we observed that twenty nine randomly selected clones from the EBV transformed cells library exhibited the VH4 and VH5 gene usage suggesting that our library may be biased towards antigen specificity. In healthy individuals, VH3 genes are most frequently used and VH5 is used only by a low percentage of antibodies (http:imgt.cines.fr). The light chain did not show any preferential gene usage and all the light chains were successfully amplified.

As heavy chain amplification was biased towards the expression of a set of heavy chain genes, to avoid predominant expression of one subset of genes in the phage library, we took an equal concentration of all the heavy chain genes which in turn increased the chances of the new combinations that are not expressed in natural infection. Successful isolation of positive clones with higher affinity and specificity is possible when a library is more diverse and has less number of clones with incomplete scFvs sequences [15,32]. If phage carrying incomplete scFvs are more in number, they overwhelm the library after multi-step panning thereby the presence of the full size insert will decrease, this being a common problem with phage libraries. Interestingly, our library exhibited 90% diversity of clones and 100% clones had complete scFvs (Figure 7A,B), this might be because of the optimum incubation time (30–40 min) maintained during the transformation process that reduced the chances of repetition of clones. In addition, the combination of the *Sfi*1*- Not*I site containing pCANTAB-5E vector that we used in the library construction may have reduced or eliminated the chances of getting incomplete scFv sequences because these restriction enzyme sites are rarely present in the scFv DNA sequence [15,33]. Furthermore, none of our clones had internal *Sfi*1*- Not*I sites.

Stringency in the biopanning also reduces the number of false positive clones. Initially, phage were allowed to bind to the plastic plate, next, to coated milk, followed by BSA and finally onto a pool of coated unrelated antigens in the plates. This stringency reduced the number of non-specific phage binders and made the screening process more convenient and specific.

Kempf et al., using a similar strategy of EBV transformation, constructed a Fab library of 10^7^ clones from which they isolated three Fabs after one round of biopanning. Their scoring of positive clones however was less, probably because they selected cells positive for gp120 binding from the 96 well stage and also included wells that were low binders ( OD> 0.1) [20]. We therefore selected only those wells with high V3 binding reactivity (OD>0.6) at all stages for the phage library construction. This reduced the number of false positive clones and enriched the V3 specific cells.

There are several reports on the generation of scFvs from different sources like PBMCs [34,35], bone marrow [36], tonsils [37], hybridomas [38,39], but none of them employed EBV transformed antigen specific cells to generate a scFv phage library.

Generation of phage library from EBV transformed lines after preselection with antigen is very useful for laboratories with limited resources because the antibody production by traditional hybridoma technology costs ranging between $8000 to $12000 and 80% of the costs are incurred in the post EBV immortalization steps like fusions and cloning. Our strategy does not require the expensive process of electroporation because the cost of cuvettes and instrument mainly limits the construction of phage libraries with such a large size and diversity; also it eliminates the large number of biopanning rounds followed by cumbersome screening processes. Such small libraries are easy to maintain and can be produced by routinely used calcium chloride mediated transformations.

## Conclusions

We have for the first time, generated anti-V3 human scFvs against clade C HIV-1. By antigen pre-selection, the phage library served as an adequate source of V3 positive clones. The combination of EBV transformation and selection of immortalized lines that exhibit the desired antigen binding characteristics with phage display technology provides a useful strategy for specific recombinant antibody generation. Our study validated the generation of recombinant libraries as a powerful tool for the generation of diverse recombinant antibodies.

## Methods

### Study subject, blood sample collection and processing

The HIV-1 seropositive patient (# 254) enrolled for the construction of the anti-V3 scFv phage library was recruited after obtaining written informed consent from the Department of Medicine, AIIMS, New Delhi. The study was approved by the institute ethics committee. Whole blood was collected in EDTA vaccutainers. The plasma was separated from whole blood, aliquoted and stored at −70°C until tested. The peripheral blood mononuclear cells (PBMCs) were separated by phycoll hypaque centrifugation and processed immediately for EBV transformation.

The plasma sample of the patient # 254 was previously tested and found to exhibit good neutralization potential against a diverse panel of viruses (Table 1). It was also tested for the presence of binding anti-V3 antibodies and the data is shown in Figure 1.

### Screening of the patient plasma (#254) for cross reactive neutralizing antibodies

The neutralization efficiency of the plasma #254was tested against a standard panel of pseudoviruses of clades A, B and C obtained from the NIH AIDS Research and Reference Reagent Program, by TZM-bl assay [40]. The standard panel of pseudoviruses has been categorized from tier 1 to tier 3, based on the decreasing order of susceptibility to neutralization by the known monoclonal antibodies [41]. The neutralization assay was carried out in 96-well tissue culture plates. Briefly, 50 μl of the heat inactivated plasma/purified scFv, at different dilutions in duplicate, was added to 200 TCID 50 of the virus and incubated for 1 h. A cell control well containing culture media only and a virus control well containing both the culture media and the virus were tested in parallel. The rest of the procedure is the same as described for calculating TCID 50. The scFv (HepB), an scFv against the Hepatitis B surface antigen [42] and 1418, a human antibody to parvovirus B19 protein, were used as negative controls. For all dilutions of test plasma, the percent neutralization was calculated based on the relative luminescence units (RLU) in the presence of plasma divided by the virus control. The cell control value was subtracted from the plasma RLU value as the background cutoff. The 50% neutralization titer (ID50 titer) was determined for the plasma sample #254 against each virus by plotting percentage neutralization against the dilution of the plasma tested. A non-linear regression straight line was drawn by the method of least squares, and the reciprocal ID50 titers were extrapolated. The experiments were performed in duplicate and repeated at least twice and the mean ID50 titers were calculated.

### Quantitation of the levels of anti-V3 Abs by Peptide ELISA

The anti-V3 Antibody content in the HIV-1 seropositive plasma sample #254 and in the supernatants of the EBV transformed PBMCs in culture at different stages was determined using V3 peptide ELISA. Thirty five mer peptides of V3C (CTRPNNNTRKSIRIGPGQTFYATGDIIGDIRQAHC) and V3B (CTRPNNNTRKSIHIGPGRAFYTTGEIIGDIRQAHC) were synthesised (Sigma Aldrich, USA), based on the consensus V3 sequences. The V3 peptides (1 μg/ml) were coated onto 96 well Nunc-Immuno plates (Nunc: Cat# 439454) using antigen coating buffer (150 mM Na_2_CO_3_, 350 mM NaHCO_3_, 30 mM NaN_3_, pH 9.6) at 4°C overnight. Plates were washed using phosphate buffered saline with 0.1% Tween-20 (0.1% PBST) thrice using a plate washer. Plates were then blocked with 100 μl of 15% fetal calf serum and incubated at 37°C for 1.5 h. Following blocking and washing, heat inactivated plasma (100 μl, dilution range=300-100000) or 100 μl of supernatants from EBV transformed PBMC cultures was added to each well and incubated for 1hour at 37°C. After 3 washings with PBST (0.1%), the bound V3 specific antibodies were detected by addition of 100 μl of alkaline-phosphatase conjugated anti-human IgG Fc (1:2000 in PBST). Immune complexes were revealed with AP-Substrate in DAE buffer and the colorimetric reaction was stopped by the addition of 6N NaOH. The optical density was read at 405 nm. ID_50_ titers were calculated for the plasma sample against each of the peptide by plotting the absorbance at 405 nm against the dilutions of the plasma sample tested. A non-linear regression straight line was drawn by the method of Least squares and the ID_50_ titers were extrapolated.

### Epstein –Barr Virus (EBV) induced transformation of PBMCs

B95 cell line comprises of EBV transformed human lymphoblastoid cells which secrete EBV in the supernatant. EBV transforms human B cells [43]. We obtained the B95 cell line from American Type Cell Culture (Cat. No. VR-1492). B95 cells were grown in T-75 tissue culture flasks, at 100,000 cells/ml at 37°C, 5% CO_2,_ in complete RPMI media. After 10 days, the viral supernatant was centrifuged at 300 × g and filtered with 0.45 μm filters. Peripheral blood mononuclear cells (PBMC) (100,000 cells/well) from the patient #254 were EBV transformed by mixing with 100 μl of the viral supernatant in a 96-well plate and cultured with a polyclonal B cell activator, CpG (2 μg/ml), which enhanced EBV infection and B cell transformation [44] and CsA (0.5 μg/ml). The plate was incubated at 37°C in a 5% CO_2_ incubator overnight. Next day, cells were fed with 100 μl of complete medium containing CsA and CpG. Cultures were fed twice per week and half of the culture supernatant was replaced with fresh complete media, 200 μl/well (no CsA and CpG).

### Screening of B-lymphocytes producing anti-V3 antibodies

After two weeks of culturing the B cells in the 96 well plate, we screened for the presence of V3 antibodies by V3 peptide binding ELISA as described above. The positive B cell clones were further expanded to 24 well plates and screened in the 3^rd^ and 4^th^ week; V3 positive clones were transferred to a six well plate, pooled and further expanded to the T-25 flask (Table 2).

### Construction of Human anti-V3 scFv phage library

Total RNA from the V3 specific antibody producing B cells (from flask stage) was isolated by Trizol reagent (Sigma, USA) and then reverse transcribed to cDNA, using the reverse aid MMuLV reverse transcriptase (Fermentas,USA). A total of 200 ng of RNA was reverse transcribed in a reaction volume of 50 μl containing, 10ng of random hexamer, 20μM oligo-dT, 1.5 μl of RNase inhibitor (40U/μl), all dissolved in 1X RT buffer. The RNA was heated to 65°C for 5 min and then immediately chilled on ice for at least five minutes. Following the addition of the reaction mixture, the tube was incubated at 42°C for 60 min, at 70°C for 5 min and quickly chilled on ice and stored in −20°C. Heavy chain variable region genes were amplified using a total of 24 combinations (6 forward primers and 4 reverse primers representing all human immunoglobulin subfamilies) and for light chain kappa, a total of 30 combinations were used (6 forward primers and 5 reverse primers) as described previously [15]. A total of 54 independent reactions were performed to generate the variable regions of heavy and light chains. The heavy chain 5^′^ primers included a *Sfi*I site and the light chain 3^′^primer included a *Not*I site. Light chain 5^′^ primer included part of the linker region (Gly_4_Ser)_3_ and this was compatible with the heavy chain 3^′^ primer. Each variable heavy region was amplified using Hot start Taq DNA polymerase (Fermentas) in a PCR reaction of 50 μl containing 2.5 μl cDNA, primers 1 μl (10 pmole each) both forward and reverse. PCR reaction was performed for 34 cycles (94°C for 3 min initial denaturation, 94°C for 1 min, annealing at 63°C for 1 min, extension at 72°C for 2 min) using eppendorf Master Cycler. Light chain variable region were amplified with the similar protocol except the annealing temperature used was 57°C. Each variable region gene was purified from the agarose gel using gel extraction kit (Qiagen, Germany). An equimolar mixture of pooled heavy and light chain DNA was used in the second round assembly PCR. The assembly PCR reaction was cycled 20 times (94°C for 1 min, 94°C for 45 sec, 62°C for 50 sec, 72°C for 2 min) the assembly reaction was performed using Pfu DNA polymerase and without primers. Full length scFvs were amplified using a pull through PCR reaction using Taq DNA polymerase and the following primers PTfw 5^′^ CCT TTC TAT GCG GCC CAG CCG GCC ATG GCC 3^′^ and PTrv 5^′^ CAG TCA TTC TCG ACT T*GC GGC CGC* ACG 3^′^ (94°C for 1 min, annealing at 62°C for 1min, extension at 72°C for 1 min and final extension at 72°C for 5 min). The scFvs were agarose gel purified using gel extraction kit (Qiagen, Germany).

### Cloning of anti-V3 scFv into pCANTAB -5E vector

The scFv DNA fragments and pCANTAB-5E vector were digested with *Not*I */*S*fi*I (New England Biolabs, USA) respectively. The digested scFv DNA fragments and pCANTAB-5E vector were gel purified using gel extraction kit (Qiagen, Germany). The scFv DNA was ligated into vector at a 3:1 molar ratio using T4 DNA ligase (New England Biolabs, USA). The ligated DNA was transformed into chemically competent cells of *E*.*coli* TG1 and placed on ice for 1h followed by heat shock treatment for 90 seconds and chilled on ice for 5 min. Next, 800 μl of 2XYT media was added and incubated in a rotating shaker at 200 rpm for 40 min. The transformed cells were plated on to 2XYT medium agar plates containing ampicillin (50 mg/ml) and 2% glucose, and incubated overnight at 37°C. The following day, colonies were scraped into 1ml of 2XYT medium with 20% glycerol and stored at −70°C. Sequence of the assembled scFv was confirmed by an automated ABI prism sequencer using gene specific primers.

### Panning of the scFv phage library

The phage were rescued by infection with helper phage (M13-KO7), followed by precipitation with PEG/NaCl, resuspension in PBS and titration for the determination of phage concentration. The phage were then subjected to a single round of enrichment by bio-panning.

The panning procedure was carried out in Immuno 96 microwell plates. Plates were coated with 100 μl of V3 peptide 1 μg/ml in 0.1 M NaHCO_3_ (pH 8.6) overnight at 4°C. A phage library of 10^12^ phage was incubated for 1h in milk coated wells to remove the non-specific binders. After one hour, unbound phage were transferred to the peptide coated wells for 30 min at RT. The unbound phage was eliminated by washing 10–15 times with PBS containing 0.1% Tween 20. The bound phage was eluted with 0.2 M glycine pH 2.2 for 10 min at RT. The eluted phage were neutralized with 1M Tris HCl pH 9.2 and immediately infected onto TG1 (OD 0 .4 to .5) for 30 min at 37°C and for 30 min with shaking at 37°C. Cells were spun down and plated on 2XTY agar containing ampicillin (50 mg/ml) and 2% glucose. Individual colonies were picked and grown in 96 well sterile culture plates (Corning) and a glycerol stock was made and stored at −70°C.

### Phage Rescue (24 well plate)

Individual colonies were grown in 1ml 2XYT broth containing ampicillin and 2% glucose overnight with shaking at 37°C at 160 rpm. A small inoculum was transferred to 1ml 2XYT broth containing ampicillin (50 mg/ml) and 2% glucose at 37°C with shaking at 200 rpm till the OD reached 0.4 to 0.5. Helper phage were added and the plate was incubated at 37°C without shaking and then for 30 min with shaking at 180 rpm at 37°C. The cells were spun down at 1500 × g, the supernatant was discarded and pellet was washed with 2XYT broth. The pellet was then resuspended in 2XYT broth containing ampicillin (50 mg/ml) and kanamycin (100 mg/ml) (no glucose) with shaking at160 rpm at 30°C for 16–18 h. It was then centrifuged at 6000 × g and the supernatant was collected and stored at 4°C and an aliquot was tested in the phage ELISA.

### Phage ELISA

The ELISA plates were coated with 100 μl of V3 peptide (1 μg/ml) in 0.1 M NaHCO_3_ (pH 8.6) and incubated overnight at 4°C. The plates were washed once with 1X PBS and blocked with 4% non-fat milk (Titan Biotech, India) for 2 h at 37°C. The plates were then washed three times with 1X PBS. Phage supernatant (100 μl) was added to each well and incubated for 1h at RT. Phage supernatant was discarded and the plates were washed four times with PBST(0.1%). 100 μl of anti M13 antibody (diluted 1:2000) was added (Sigma) and incubated for 1h at RT. The plates were washed four times with PBST (0.1%). 100 μl of anti rabbit HRP (Jackson) diluted 1:3000 were added and incubated at RT for 1h. The plates were then washed four times with PBST (0.1%). 100 μl of TMB substrate was added, and the reaction was stopped by adding 8N H_2_SO_4._ Absorbance was measured at 450nm.

### PEG precipitation of phage (pure phage preparation)

Individual bacterial colonies were grown in 5ml 2XYT medium containing ampicillin (50 mg/ml) and 2% glucose with shaking at 200 rpm at 37°C till the O.D reached 0.4 to 0.5. Next, 1 μl of KO7 helper phage (10^18^) was added and incubated at 37°C for 30 min without shaking, followed by 30 min shaking at 200 rpm. The cells were centrifuged at 2500 × g for 10 min, supernatant was discarded and the pellet was washed again with 2XYT broth. The pellet was resuspended in 50ml 2XYT broth containing Ampicillin (50 mg/ml) and Kanamycin (100 mg/ml) (no glucose) at 30°C with shaking at 160 rpm for 12–16 h. It was then centrifuged at 10000 × g for 20 min at 4°C. The supernatant was transferred to glass bottles and Â¼ volume of PEG/NaCl was added and kept on ice for 4 to 6 h followed by centrifugation at 20000 × g for 20 min at 4°C. The supernatant was discarded and the pellet was dissolved in sterile and autoclaved 1X PBS and stored at 4°C. The transformation unit (TU) was calculated.

### DNA sequencing and sequence analysis

Twenty nine scFvs clones were randomly selected from the unselected library and sequenced by Macrogen (South Korea). The sequences were analysed using immunoglobulin BLAST [45] and V BASE software [46].

### DNA fingerprinting of antibody fragments

The diversity of the scFv repertoire was analysed by comparing the restriction digestion pattern of scFvs. Ten clones were randomly selected from the primary phage library and the plasmid was isolated. The scFv sequences were amplified using primers PTfw 5^′^ CCT TTC TAT GCG GCC CAG CCG GCC ATG GCC 3^′^ and PTrv 5^′^ CAG TCA TTC TCG ACT T*GC GGC CGC* ACG 3^′^ (94°C for 1 min, annealing at 62°C for 1min, extension at 72°C for 1 min and final extension at 72°C for 5 min). The amplified PCR products were digested with a frequent cutter restriction enzyme B*stN*1 (NEB) and analysed on 2% agarose gel.

### Soluble scFv expression

Clones showing positivity/binding in phage ELISA were selected for soluble scFv expression. These were than transformed into HB2151 using calcium chloride mediated transformation for soluble scFv expression*.* The HB2151 cells carrying pCANTA-5E plasmid were grown in 10 ml 2XTY medium overnight at 37°C with shaking at 200 rpm. The next day, 1/100^th^ volume of the overnight culture was inoculated in 1 litre of 2XTY medium and grown at 37°C with shaking at 240 rpm till the OD reached 0.6 and then the culture was induced by 1 mM IPTG for 6 to 8 h at 24°C [33]. Cells were harvested and different fractions were prepared. scFvs were purified from the periplasmic fraction. Briefly, the cells were harvested by centrifugation at 4000 × g for 15 min at 4°C. The supernatant was discarded and pellet was resuspended in 30 mM Tris-Cl. 20% sucrose, pH 8.0 at 80 ml/ gram wet weight. The cells were placed on ice for 20 min and 500 mM EDTA was added to a final concentration of 1 mM EDTA. The cells were spun down at 8000 × g for 15 min at 4°C. The pellet was resuspended in 5 mM MgSO_4_ and the cells were placed on ice for 10 min with slowly stirring, pelleted down at 8000 × g for 15 min at 4°C and the supernatant collected for purification.

### Purification of E-tagged scFv

Purification of E-tagged scFv was carried out using the Recombinant Phage Antibody System (RPAS) Purification Module (Amersham Biosciences) as per the manufacturer’s instructions. The periplasmic extract was filtered through a 0.45 μm filter to remove any remaining cell debris. The Anti-E Tag column was regenerated by washing the column with 15ml of elution buffer (0.1 M glycine, pH 3.0) and was then equilibrated with 25 ml binding buffer (0.02 M phosphate buffer, 0.005% NaN_3_, pH 7.0). The E-tagged scFv in the periplasmic extract was then allowed to bind to the column by passing the extract through the column. The unbound excess *E. coli* proteins were removed from the column by washing it with 25 ml binding Buffer. The flow rate at each step was maintained at 5 ml/min through the column. Finally, the bound scFv was eluted by 15ml elution buffer. Several fractions of the eluted scFv (900 μl each) were collected in tubes containing 100 μl neutralization buffer (1 M Tris, 0.05% NaN3, pH 8.2). The amount of protein in each fraction was estimated using BCA method and the fractions containing considerable amount of scFv were pooled together and concentrated.

### Soluble ELISA

Soluble ELISA was performed, as described in the phage ELISA. Hundred microliter of soluble scFv periplasmic extract/purified scFv was added and incubated at room temperature for 1h. ELISA plates were washed three times with 0.1% PBST and incubated with 1:1000 dilution of primary antibody in 2% MPBS. The plates were washed three times with 0.1% PBST and incubated with 1:2000 diluted anti rabbit HRP conjugated secondary antibody in 2% MPBS. The ELISA plates were washed again as described above. 100 μl of TMP substrate was added and incubated at RT till the colour developed. Reaction was stopped by adding 8NH_2_SO_4._ Absorbance was read at 450 nm.

### SDS-PAGE and Western blot

SDS-PAGE was done as described [33]. Proteins were separated on a 12% running gel and 5% stacking gel and visualized by Coomassie Brilliant Blue (CBB) staining. For Western blotting, gel was blotted onto nitrocellulose membrane using electroblotting, (100 V for 1h) and probed with primary antibody. Anti-rabbit HRP was used as secondary antibody and colour was developed with DAB as the substrate.

## Competing interests

The authors declare that they have no competing interests.

## Authors’ contributions

KL and SS designed and conceptualized the study and finalized the manuscript. RK constructed the phage library, constructed the scFvs and wrote the manuscript. RA screened the plasma of patients for neutralization and performed the EBV transformation experiments. AT provided valuable inputs in phage library construction. SSP, DD,AS and LK helped in protein expression, purification and phage ELISA. NW provided the patient samples. All authors have read and approved the final manuscript.

## References

[B1] Moulard M Phogat SK Shu Y Labrijn AF Xiao X Binley JM Zhang M-Y Sidorov IA Broder CC Robinson J Parren PWHI Burton DR Dimitrov DS Broadly cross-reactive HIV-1-neutralizing human monoclonal Fab selected for binding to gp120-CD4-CCR5 complexes Proc Natl Acad Sci USA 2002 99 6913 6918 10.1073/pnas.102562599 11997472 PMC124503

[B2] Zolla-Pazner S Zhong P Revesz K Volsky B Williams C Nyambi P Gorny MK The cross-clade neutralizing activity of a human monoclonal antibody is determined by the GPGR V3 motif of HIV type 1 AIDS Res Hum Retroviruses 2004 20 1254 1258 10.1089/aid.2004.20.1254 15588347

[B3] Burton DR Pyati J Koduri R Sharp SJ Thornton GB Parren PW Sawyer LS Hendry RM Dunlop N Nara PL Efficient neutralization of primary isolates of HIV-1 by a recombinant human monoclonal antibody Science 1994 266 1024 1027 10.1126/science.7973652 7973652

[B4] Gorny MK Williams C Volsky B Revesz K Wang X-H Burda S Kimura T Konings FAJ Nádas A Anyangwe CA Nyambi P Krachmarov C Pinter A Zolla-Pazner S Cross-clade neutralizing activity of human anti-V3 monoclonal antibodies derived from the cells of individuals infected with non-B clades of human immunodeficiency virus type 1 J Virol 2006 80 6865 6872 10.1128/JVI.02202-05 16809292 PMC1489067

[B5] Pantophlet R Ollmann Saphire E Poignard P Parren PWHI Wilson IA Burton DR Fine mapping of the interaction of neutralizing and nonneutralizing monoclonal antibodies with the CD4 binding site of human immunodeficiency virus type 1 gp120 J Virol 2003 77 642 658 10.1128/JVI.77.1.642-658.2003 12477867 PMC140633

[B6] Posner MR Hideshima T Cannon T Mukherjee M Mayer KH Byrn RA An IgG human monoclonal antibody that reacts with HIV-1/GP120, inhibits virus binding to cells, and neutralizes infection J Immunol 1991 146 4325 4332 1710248

[B7] Trkola A Dragic T Arthos J Binley JM Olson WC Allaway GP Cheng-Mayer C Robinson J Maddon PJ Moore JP CD4-dependent, antibody-sensitive interactions between HIV-1 and its co-receptor CCR-5 Nature 1996 384 184 187 10.1038/384184a0 8906796

[B8] Scheid JF Mouquet H Feldhahn N Seaman MS Velinzon K Pietzsch J Ott RG Anthony RM Zebroski H Hurley A Phogat A Chakrabarti B Li Y Connors M Pereyra F Walker BD Wardemann H Ho D Wyatt RT Mascola JR Ravetch JV Nussenzweig MC Broad diversity of neutralizing antibodies isolated from memory B cells in HIV-infected individuals Nature 2009 458 636 640 10.1038/nature07930 19287373

[B9] Walker LM Phogat SK Chan-Hui P-Y Wagner D Phung P Goss JL Wrin T Simek MD Fling S Mitcham JL Lehrman JK Priddy FH Olsen OA Frey SM Hammond PW Kaminsky S Zamb T Moyle M Koff WC Poignard P Burton DR Broad and potent neutralizing antibodies from an African donor reveal a new HIV-1 vaccine target Science 2009 326 285 289 10.1126/science.1178746 19729618 PMC3335270

[B10] Wu X Yang Z-Y Li Y Hogerkorp C-M Schief WR Seaman MS Zhou T Schmidt SD Wu L Xu L Longo NS McKee K O’Dell S Louder MK Wycuff DL Feng Y Nason M Doria-Rose N Connors M Kwong PD Roederer M Wyatt RT Nabel GJ Mascola JR Rational design of envelope identifies broadly neutralizing human monoclonal antibodies to HIV-1 Science 2010 329 856 861 10.1126/science.1187659 20616233 PMC2965066

[B11] Gray ES Meyers T Gray G Montefiori DC Morris L Insensitivity of paediatric HIV-1 subtype C viruses to broadly neutralising monoclonal antibodies raised against subtype B PLoS Med 2006 3 e255 10.1371/journal.pmed.0030255 16834457 PMC1502151

[B12] Moore PL Gray ES Sheward D Madiga M Ranchobe N Lai Z Honnen WJ Nonyane M Tumba N Hermanus T Sibeko S Mlisana K Abdool Karim SS Williamson C Pinter A Morris L Potent and broad neutralization of HIV-1 subtype C by plasma antibodies targeting a quaternary epitope including residues in the V2 loop J Virol 2011 85 3128 3141 10.1128/JVI.02658-10 21270156 PMC3067856

[B13] Gorny MK Gianakakos V Sharpe S Zolla-Pazner S Generation of human monoclonal antibodies to human immunodeficiency virus Proc Natl Acad Sci USA 1989 86 1624 1628 10.1073/pnas.86.5.1624 2922401 PMC286751

[B14] Smith GP Filamentous fusion phage: novel expression vectors that display cloned antigens on the virion surface Science 1985 228 1315 1317 10.1126/science.4001944 4001944

[B15] Pansri P Jaruseranee N Rangnoi K Kristensen P Yamabhai M A compact phage display human scFv library for selection of antibodies to a wide variety of antigens BMC Biotechnol 2009 9 6 10.1186/1472-6750-9-6 19175944 PMC2642811

[B16] Winter G Griffiths AD Hawkins RE Hoogenboom HR Making antibodies by phage display technology Annu Rev Immunol 1994 12 433 455 10.1146/annurev.iy.12.040194.002245 8011287

[B17] Bugli F Graffeo R Paroni Sterbini F Torelli R Masucci L Sali M Grasso A Rufini S Ricci E Fadda G Pescatori M Monoclonal antibody fragment from combinatorial phage display library neutralizes alpha-latrotoxin activity and abolishes black widow spider venom lethality, in mice Toxicon 2008 51 547 554 10.1016/j.toxicon.2007.11.014 18187177

[B18] Andre F Fröde D Meyer T Schirrmann T Hust M Generating Recombinant Antibodies for Research, Diagnostics and Therapy Using Phage Display Current Biotechnology 2012 1 33 41 10.2174/2211550111201010033

[B19] Kuwata T Katsumata Y Takaki K Miura T Igarashi T Isolation of potent neutralizing monoclonal antibodies from an SIV-Infected rhesus macaque by phage display AIDS Res Hum Retroviruses 2011 27 487 500 20854170 10.1089/AID.2010.0191

[B20] Kempf E Weiss E Klein P Glacet A Spratt S Bourel D Orfanoudakis G The rescue by phage display of human Fabs to gp120 HIV-1 glycoprotein using EBV transformed lymphocytes Mol Biotechnol 2001 17 97 108 10.1385/MB:17:2:097 11395866

[B21] Hemelaar J Gouws E Ghys PD Osmanov S Global and regional distribution of HIV-1 genetic subtypes and recombinants in 2004 AIDS 2006 20 W13 W23 10.1097/01.aids.0000247564.73009.bc 17053344

[B22] Cardozo T Kimura T Philpott S Weiser B Burger H Zolla-Pazner S Structural basis for coreceptor selectivity by the HIV type 1 V3 loop AIDS Res Hum Retroviruses 2007 23 415 426 10.1089/aid.2006.0130 17411375

[B23] Sharon M Kessler N Levy R Zolla-Pazner S Görlach M Anglister J Alternative conformations of HIV-1 V3 loops mimic beta hairpins in chemokines, suggesting a mechanism for coreceptor selectivity Structure 2003 11 225 236 10.1016/S0969-2126(03)00011-X 12575942

[B24] Hill CM Deng H Unutmaz D Kewalramani VN Bastiani L Gorny MK Zolla-Pazner S Littman DR Envelope glycoproteins from human immunodeficiency virus types 1 and 2 and simian immunodeficiency virus can use human CCR5 as a coreceptor for viral entry and make direct CD4-dependent interactions with this chemokine receptor J Virol 1997 71 6296 6304 9261346 10.1128/jvi.71.9.6296-6304.1997 PMC191902

[B25] Wang WK Dudek T Essex M Lee TH Hypervariable region 3 residues of HIV type 1 gp120 involved in CCR5 coreceptor utilization: therapeutic and prophylactic implications Proc Natl Acad Sci USA 1999 96 4558 4562 10.1073/pnas.96.8.4558 10200301 PMC16371

[B26] Andrabi R Choudhary AK Bala M Kalra R Prakash SS Pandey RM Luthra K Relative reactivity of HIV-1 polyclonal plasma antibodies directed to V3 and MPER regions suggests immunodominance of V3 over MPER and dependence of high anti-V3 antibody titers on virus persistence Arch Virol 2011 156 1787 1794 10.1007/s00705-011-1053-5 21735212

[B27] Andrabi R Bala M Kumar R Wig N Hazarika A Luthra K Neutralization of tier-2 viruses and epitope profiling of plasma antibodies from human immunodeficiency virus type 1 infected donors from India PLoS One 2012 7 e43704 10.1371/journal.pone.0043704 22952740 PMC3432049

[B28] Krebber A Bornhauser S Burmester J Honegger A Willuda J Bosshard HR Plückthun A Reliable cloning of functional antibody variable domains from hybridomas and spleen cell repertoires employing a reengineered phage display system J Immunol Methods 1997 201 35 55 10.1016/S0022-1759(96)00208-6 9032408

[B29] Gorny MK Sampson J Li H Jiang X Totrov M Wang X-H Williams C O’Neal T Volsky B Li L Cardozo T Nyambi P Zolla-Pazner S Kong X-P Human anti-V3 HIV-1 monoclonal antibodies encoded by the VH5-51/VL lambda genes define a conserved antigenic structure PLoS One 2011 6 e27780 10.1371/journal.pone.0027780 22164215 PMC3229485

[B30] David D Goossens D Desgranges C Thèze J Zouali M Molecular characterization of human monoclonal antibodies specific for several HIV proteins: analysis of the VH3 family expression Immunol Lett 1995 47 107 112 10.1016/0165-2478(95)00078-J 8537086

[B31] Wisnewski A Cavacini L Posner M Human antibody variable region gene usage in HIV-1 infection J Acquir Immune Defic Syndr Hum Retrovirol 1996 11 31 38 10.1097/00042560-199601010-00004 8528730

[B32] Chahboun S Hust M Liu Y Pelat T Miethe S Helmsing S Jones RG Sesardic D Thullier P Isolation of a nanomolar scFv inhibiting the endopeptidase activity of botulinum toxin A, by single-round panning of an immune phage-displayed library of macaque origin BMC Biotechnol 2011 11 113 10.1186/1472-6750-11-113 22111995 PMC3252318

[B33] Burmester J Spinelli S Pugliese L Krebber A Honegger A Jung S Schimmele B Cambillau C Plückthun A Selection, characterization and x-ray structure of anti-ampicillin single-chain Fv fragments from phage-displayed murine antibody libraries J Mol Biol 2001 309 671 685 10.1006/jmbi.2001.4663 11397088

[B34] Rangnoi K Jaruseranee N O’Kennedy R Pansri P Yamabhai M One-step detection of aflatoxin-B(1) using scFv-alkaline phosphatase-fusion selected from human phage display antibody library Mol Biotechnol 2011 49 240 249 10.1007/s12033-011-9398-2 21465334

[B35] Little M Welschof M Braunagel M Hermes I Christ C Keller A Rohrbach P Kürschner T Schmidt S Kleist C Terness P Generation of a large complex antibody library from multiple donors J Immunol Methods 1999 231 3 9 10.1016/S0022-1759(99)00164-7 10648923

[B36] Okamoto T Mukai Y Yoshioka Y Shibata H Kawamura M Yamamoto Y Nakagawa S Kamada H Hayakawa T Mayumi T Tsutsumi Y Optimal construction of non-immune scFv phage display libraries from mouse bone marrow and spleen established to select specific scFvs efficiently binding to antigen Biochem Biophys Res Commun 2004 323 583 591 10.1016/j.bbrc.2004.08.131 15369791

[B37] Vaughan TJ Williams AJ Pritchard K Osbourn JK Pope AR Earnshaw JC McCafferty J Hodits RA Wilton J Johnson KS Human antibodies with sub-nanomolar affinities isolated from a large non-immunized phage display library Nat Biotechnol 1996 14 309 314 10.1038/nbt0396-309 9630891

[B38] Bose B Chugh DA Kala M Acharya SK Khanna N Sinha S Characterization and molecular modeling of a highly stable anti-Hepatitis B surface antigen scFv Mol Immunol 2003 40 617 631 10.1016/j.molimm.2003.07.002 14597165

[B39] Zhang MY Shu Y Rudolph D Prabakaran P Labrijn AF Zwick MB Lal RB Dimitrov DS Improved breadth and potency of an HIV-1-neutralizing human single-chain antibody by random mutagenesis and sequential antigen panning J Mol Biol 2004 335 209 219 10.1016/j.jmb.2003.09.055 14659751

[B40] Montefiori DC Measuring HIV neutralization in a luciferase reporter gene assay Methods Mol Biol 2009 485 395 405 10.1007/978-1-59745-170-3_26 19020839

[B41] Seaman MS Janes H Hawkins N Grandpre LE Devoy C Giri A Coffey RT Harris L Wood B Daniels MG Bhattacharya T Lapedes A Polonis VR McCutchan FE Gilbert PB Self SG Korber BT Montefiori DC Mascola JR Tiered categorization of a diverse panel of HIV-1 Env pseudoviruses for assessment of neutralizing antibodies J Virol 2010 84 1439 1452 10.1128/JVI.02108-09 19939925 PMC2812321

[B42] Tiwari A Khanna N Acharya SK Sinha S Humanization of high affinity anti-HBs antibody by using human consensus sequence and modification of selected minimal positional template and packing residues Vaccine 2009 27 2356 2366 10.1016/j.vaccine.2009.02.019 19428851

[B43] Caputo JL Thompson A McClintock P Reid YA Hay RJ An effective method for establishing human B lymphoblastic cell lines using epstein-barr virus Methods Cell Sci 1991 13 39 44

[B44] Traggiai E Becker S Subbarao K Kolesnikova L Uematsu Y Gismondo MR Murphy BR Rappuoli R Lanzavecchia A An efficient method to make human monoclonal antibodies from memory B cells: potent neutralization of SARS coronavirus Nat Med 2004 10 871 875 10.1038/nm1080 15247913 PMC7095806

[B45] NCBI Ig BLASThttp://www.ncbi.nlm.nih.gov/igblast/

[B46] Althaus H-H Müller W Tomlinson I V BASEhttp://vbase.mrccpe.cam.ac.uk/

